# Aggregation and Propagation of α‐Synuclein in Parkinson's Disease: A Bibliometric Perspective

**DOI:** 10.1002/brb3.71023

**Published:** 2025-10-29

**Authors:** Xinyue Zhang, Yizhao Ma, Ge Gao, Qihui Wu

**Affiliations:** ^1^ Shanghai Key Laboratory of Anesthesiology and Brain Functional Modulation, Clinical Research Center for Anesthesiology and Perioperative Medicine, Translational Research Institute of Brain and Brain‐Like Intelligence, Shanghai Fourth People's Hospital Affiliated to Tongji University School of Medicine, Shanghai Research Institute for Intelligent Autonomous Systems, State Key Laboratory of Cardiology and Medical Innovation Center, Shanghai East Hospital, School of Medicine Tongji University Shanghai China; ^2^ Center For Translational Neurodegeneration and Regenerative Therapy Tongji Hospital Affiliated to Tongji University School of Medicine Shanghai China

**Keywords:** aggregation, α‐synuclein, bibliometric analysis, Parkinson's disease, propagation

## Abstract

**Introduction:**

Parkinson's disease (PD) is a progressive neurodegenerative disorder characterized by the aggregation and propagation of alpha‐synuclein (α‐syn), processes that contribute to neuronal dysfunction and cell death. Although substantial progress has been made in understanding α‐syn pathology, a comprehensive bibliometric evaluation of global trends in this field remains lacking. This study aims to systematically map the research landscape surrounding α‐syn aggregation and propagation in PD, offering insights into its molecular mechanisms and clinical relevance.

**Methods:**

A bibliometric analysis was conducted using data retrieved from the Web of Science Core Collection (WoSCC) spanning 2005 to 2024. The data were processed and analyzed with R‐Bibliometrix, VOSviewer, and CiteSpace to quantify publication trends, international collaborations, influential authors and institutions, journal impact, and keyword co‐occurrence networks.

**Results:**

A total of 3220 relevant articles were identified. The number of annual publications steadily increased, reflecting growing scholarly interest. The United States, China, and the United Kingdom emerged as leading contributors. Robust international collaborations were observed, especially among Western countries, with the University of California System identified as the most prolific institution. Two primary thematic clusters were revealed: (1) “aggregation,” focusing on the roles of “mitochondria” and “lysosomes,” and (2) “propagation,” highlighting the involvement of “exosomes” and “microglia.” Emerging research frontiers included “biomarkers” and “neuroinflammation,” with a recent trend shifting toward studies on the propagation of α‐syn.

**Conclusion:**

This study underscores a paradigm shift in PD research from α‐syn aggregation to propagation, emphasizing the significance of exosomes, microglia, and systemic inflammation in disease pathogenesis. These findings provide a comprehensive roadmap for future research, highlighting the need for interdisciplinary collaboration and the development of targeted therapeutic strategies.

## Graphical abstract

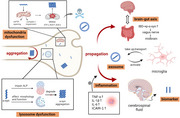

**Abbreviations**
ALP: autophagosome‐lysosome fusion pathway
APY: average publication year
ATP: adenosine triphosphate
ATPase: adenosine triphosphate synthase
CNS: central nervous system
CSF: cerebrospinal fluid
GCase: glucocerebrosidase
IBD: inflammatory bowel disease
ICAM: intercellular adhesion molecule
IL: interleukin
LBs: Lewy bodies
LPS: lipopolysaccharide
MCP: multiple country publications
mPTP: mitochondria permeability transition pore
NP: number of publications
OB: olfactory bulb
PD: Parkinson's disease
PNS: peripheral nervous system
ROS: reactive oxygen species
SCP: single country publications
SOD: superoxide dismutase
TC: total citations
TLRs: toll‐like receptors
TLS: total link strength
TNF: tumor necrosis factor
α‐Gal A: α‐galactosidase A
α‐syn: alpha‐synuclein

## 1 Introduction

Parkinson's disease (PD) is the second most prevalent neurodegenerative disorder globally, primarily characterized by motor symptoms such as tremor, rigidity, bradykinesia, and postural instability(Jankovic 2008). The pathological hallmarks of PD include the progressive degeneration of dopaminergic neurons in the substantia nigra pars compacta (SNpc) and the accumulation of Lewy bodies (LBs) (Balestrino and Schapira 2020). Alpha‐synuclein (α‐syn), a 140‐amino‐acid protein abundantly expressed in the brain, is the primary constituent of LBs. It is predominantly localized in neurons, especially at synaptic terminals, where it plays a critical role in PD progression through multiple mechanisms (Schweighauser et al. 2020). In recent years, the aggregation and propagation of α‐syn have attracted considerable attention.

The aggregation of α‐syn is recognized as a central pathogenic mechanism in PD (Baba et al. 1998). Under physiological conditions, α‐syn functions as a soluble presynaptic protein involved in vesicle trafficking and neurotransmitter release. However, under pathological conditions, post‐translational modifications expose its hydrophobic core, leading to amyloidogenic aggregation. Initially, soluble α‐syn oligomers are formed, which exhibit potent neurotoxicity and have been detected not only in neurons but also in blood and cerebrospinal fluid (Lobanova et al. 2022; Mehra, Sahay, and Maji 2019). These oligomers can further assemble into protofibrils, which eventually mature into amyloid fibrils with characterized by β‐sheet‐rich structures and cross‐β conformations, ultimately depositing as LBs (Choong and Mochizuki 2022). The presence of LBs may disrupt neuronal function by acting as space‐occupying inclusions and interfering with essential cellular processes.

Furthermore, pathological α‐syn aggregates are capable of “prion‐like” propagation (Brundin et al. 2008). This intercellular transmission occurs through multiple pathways, including endocytosis, direct membrane penetration, exosomes release, tunneling nanotubes, and receptor‐mediated uptake (Angot and Brundin 2009). α‐syn can propagate directly between neurons or indirectly via glial cells to other neurons (Xia et al. 2019). On one hand, α‐syn propagation facilitates the spreading of misfolded proteins across various brain regions, worsening central nervous system (CNS) pathology. It propagates via the vagus nerve to peripheral organs such as the gastrointestinal tract, skin, and heart, contributing to multi‐system atrophy (Braak et al. 2002; Wakabayashi 2020). On the other hand, α‐syn may also retrogradely transported from the peripheral nervous system (PNS) to the CNS through the gut‐brain axis, potentially initiating the development of PD (Mulak and Bonaz 2015).

Bibliometric analysis is a research methodology based on mathematical and statistical principles, designed to systematically evaluate the research trends, knowledge structure, academic influence, and emerging hotspots within a specific scientific domain (Ellegaard and Wallin 2015). By quantitatively analyzing data such as publication volume, citation patterns, author collaboration networks, and keyword co‐occurrence, bibliometric analysis uncovers the developmental trajectory of a field, identifies key contributors, and suggests future research directions. Compared to traditional narrative reviews, bibliometric approaches offer broader coverage, higher efficiency, and enhanced objectivity, thereby minimizing the influence of individual authors' academic backgrounds and cognitive biases (Donthu et al. 2021). Although numerous reviews have explored the roles of α‐syn aggregation and propagation in PD relatively, few studies have employed objective bibliometric approaches.

Therefore, this study aims to apply bibliometric analysis to investigate research trends and emerging focal points related to α‐syn aggregation and propagation in PD, providing novel insights from a neuropathological perspective to inform the development of therapeutic strategies.

## 2 Materials and Methods

**FIGURE 1:**
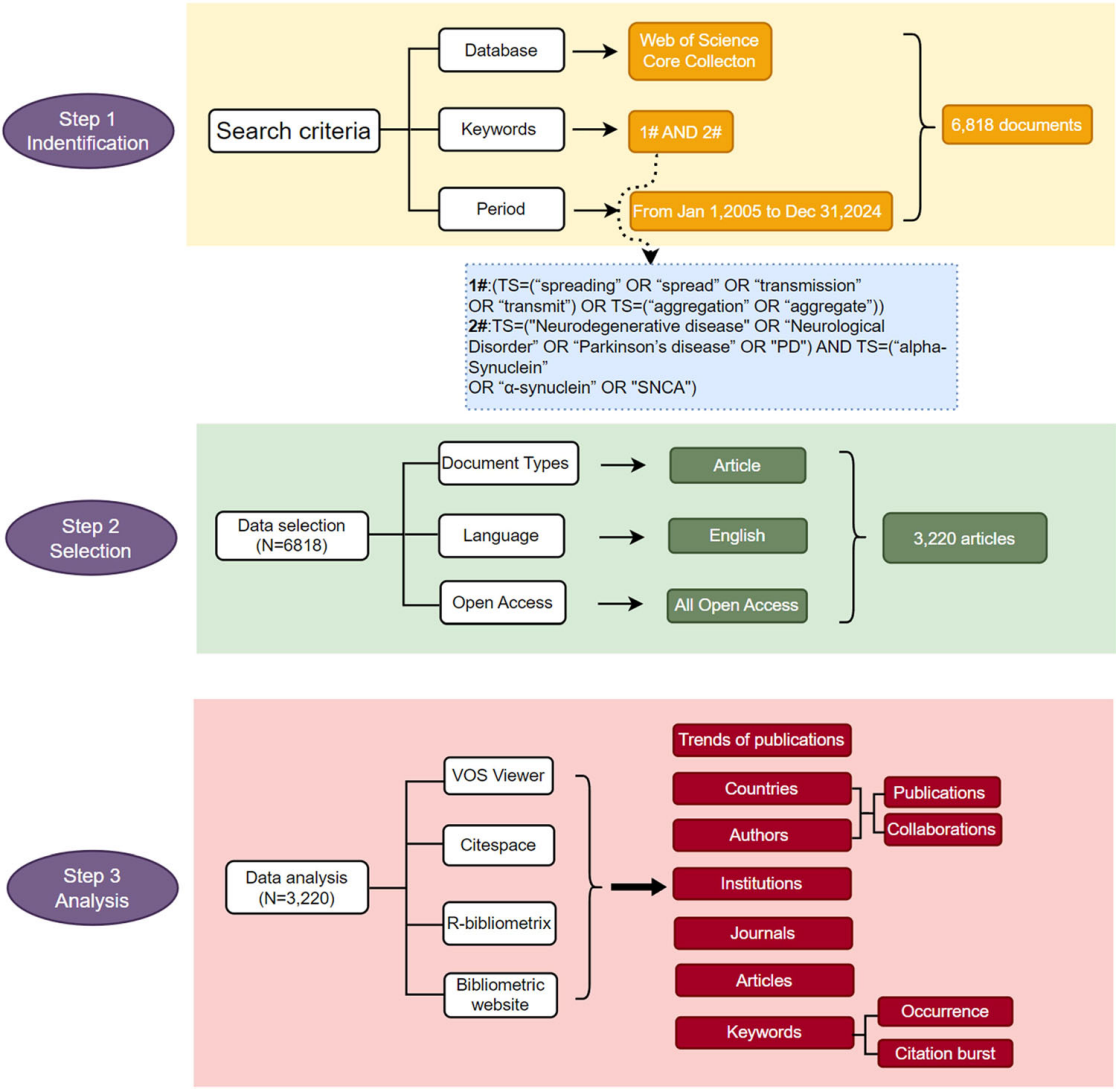
Schematic representation of data collection and study design process.

The methodological workflow comprised three major steps: initial data identification based on rigorous search criteria, careful document selection to ensure data quality, and bibliometric analysis using multiple specialized tools. This process facilitated a comprehensive exploration of research trends and formed the basis for subsequent discussion (Figure 1).

### 2.1 Data Source and Search Strategy

Relevant literature was retrieved from the Web of Science Core (WoSCC). To minimize inconsistencies due to the rapid updates of indexed articles, all data were collected on a single day. The search strategy was defined as follows: (TS = (“spreading” OR “spread” OR “transmission” OR “transmit”) OR TS = (“aggregation” OR “aggregate”)) AND TS = (“Neurodegenerative disease” OR “Neurological Disorder” OR “Parkinson's disease” OR “PD”) AND TS = (“alpha‐Synuclein” OR “α‐synuclein” OR “SNCA”). The search covered the time period from January 1, 2005 to December 31, 2024, yielding 6818 records. To enhance the accuracy of the analysis, only English‐language original research articles were included. The dataset was further restricted to “All Open Access” publications to ensure data availability and reproducibility. Finally, a total of 3220 articles were collected for the subsequent analysis.

### 2.2 Data Validation and Statistical Analysis

A bibliometric approach was utilized to assess research trends related to α‐synuclein aggregation and propagation in PD, both quantitatively and qualitatively. Date was analyzed using R‐Bibliometrix version (4.4.2), the bibliometric online platform (https://bibliometric.com/), VOSviewer (version 1.6.20), and CiteSpace software (6.1.R2).

R‐Bibliometrix is a comprehensive R‐based analytical framework that facilitates advanced quantitative analysis of scientific literature (Aria and Cuccurullo 2017). Developed as part of the Bibliometrix package, it integrates statistical precision with the flexibility of the R programming environment, enabling in‐depth exploration of publication trends, collaboration networks, and conceptual structures.

The bibliometric online platform as a complementary tool for visualizing international collaboration networks, particularly among countries, enhancing the interpretability of R‐Bibliometrix output.

VOSviewer, developed by the Centre for Science and Technology Studies (CWTS) at Leiden University, is a widely used software tool for constructing and visualizing bibliometric networks (van Eck and Waltman 2010). It enables the creation interactive, cluster‐based maps representing co‐authorship, international collaboration, and keyword co‐occurrence. In these visualizations, nodes represent elements such as countries, authors, or keywords. Node size reflects publication count or keyword frequency, while color denotes cluster membership. Total link strength (TLS), indicated by the thickness of connecting lines, quantifies the intensity of relationships between nodes. Additionally, the Average Publication Year (APY) is represented via a color gradient ranging from blue (earlier years) to yellow (more recent publications), allowing temporal analysis of emerging trends.

CiteSpace is a Java‐based tool specifically designed for visualizing and analyzing trends and patterns in scientific literature. It is usually utilized for discovering the strongest citation bursts of keywords within a period. Therefore, hotspots of a specific domain can be deduced from the results (Chen 2006).

### 2.3 Risk of Bias (Quality) Assessment

Three researchers participated in this process. Two researchers independently extracted keywords from the final set of articles and got the same number of keywords. Subsequently, these two researchers identified synonymous terms and standardized expressions using the PubMed MeSH database, respectively, and jointly carried out the final analysis of this study. Upon reaching an agreement on the final expression of the terms, they independently made revisions and removed duplicates. In cases of discrepancies between the results of these two researchers, the third researcher was consulted for a final decision.

## 3 Results

### 3.1 Trends in Annual Publications and Citations

**FIGURE 2:**
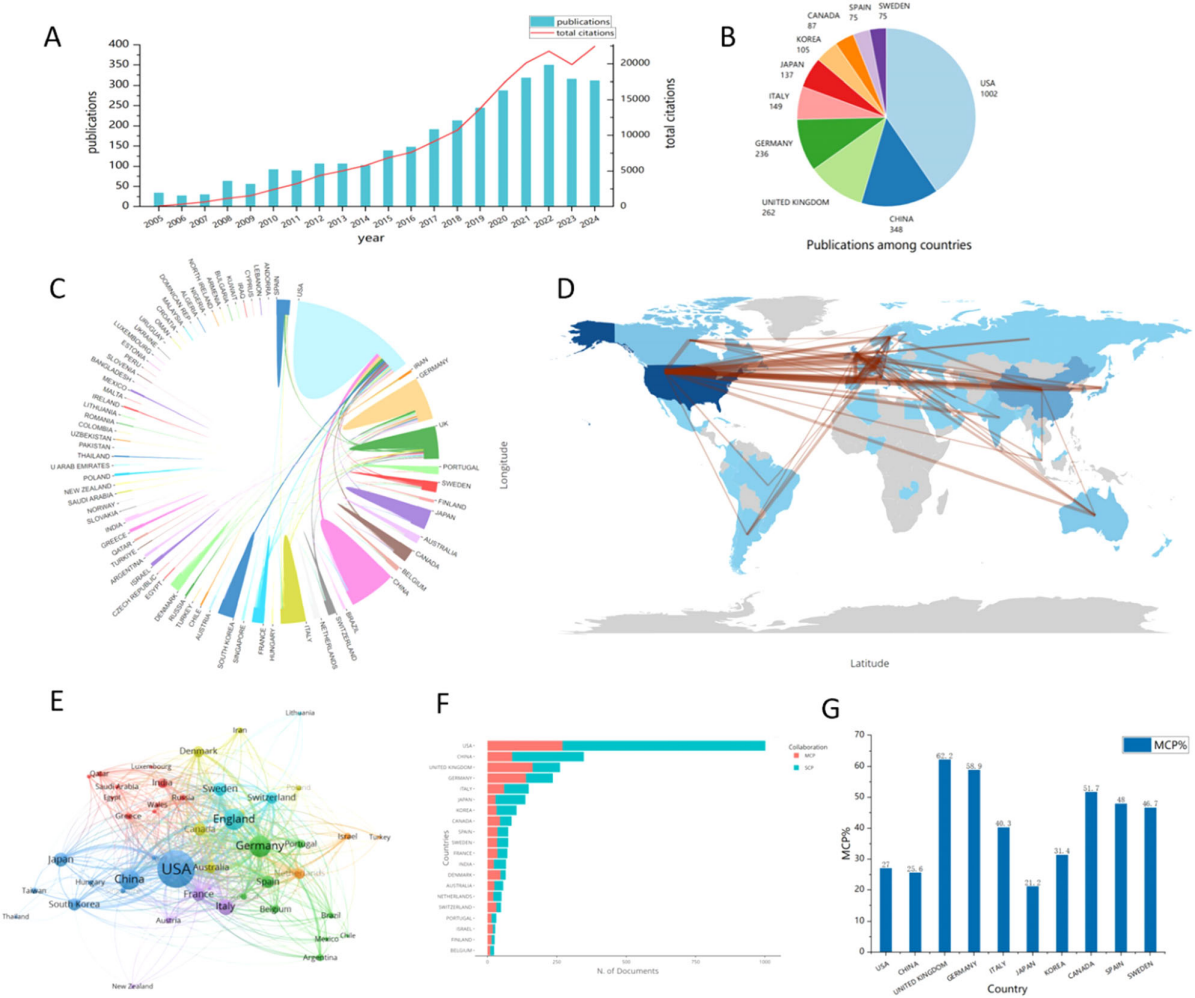
Analysis of publications and countries. (A) Trends of annual publications and total citations regarding aggregation or spread of α‐syn in PD through years. (B) Publications of the top 10 countries. (C) Collaborative research between countries. (D) Map of multinational collaborations. (E) Map of international collaboration work. (F) Distribution of corresponding author's country. MCP means the number of papers co‐authored with different countries, and SCP means the number of papers co‐authored with same countries. (G) The ratio of MCP%.

A total of 3220 articles published between 2005 and 2024 were identified and included in the analysis. The annual number of publications and total citations over this period is presented in Figure 2A. Despite minor fluctuations, there was a clear upward trend in annual publication output, increasing from 27 articles in 2006 to a peak of 341 in 2022. This growth indicates a steadily rising interest in the study of α‐syn aggregation and propagation in PD. Concurrently, the increasing number of total citations over time reflects the growing recognition and influence of this research field.

### 3.2 Distribution of Countries

The distribution of publications among the top 10 contributing countries is illustrated in a pie chart (Figure 2B). The leading countries in terms of publication volume were the United States (1002), China (348), the United Kingdom (262), Germany (236), Italy (149), Japan (137), South Korea (105), Canada (87), Spain (75), and Sweden (75). Among these, the United States held a dominant position, contributing nearly one‐third of the total publications, this predominance is likely attributable to its strong research infrastructure and substantial funding support.

### 3.3 Collaboration Among Countries

International collaborations in this field were visualized using a chord diagram and a world map (Figure 2C and D). The map showed that countries engaged in research in this field were distributed all over the world. The brown lines represented collaborative relationships between countries, with the thickness of the lines indicating the frequency of cooperation. The lines radiating from the United States demonstrated its extensive collaboration with other nations. A national collaboration network illustrated these cooperative relationships (Figure 2E). In this visualization, the node size corresponded to the number of publications, while the thickness of connecting lines denoted the frequency of collaboration. Overall, the United States exhibited the highest level of international collaboration, most notably with Germany, followed by the United Kingdom. The United States‐China partnership ranked third in terms of collaboration frequency.

However, due to the high total publication output of the United States, the frequency of collaboration alone may not accurately reflect its collaborative inclination. To address this, the Multiple Country Publications (MCP) and Single Country Publications (SCP) were introduced. MCP% represents the proportion of a country's total publications that were co‐authored with researchers from other countries. The MCP, SCP, and MCP% of the top 10 most productive countries are visualized (Figure 2F and G). The results demonstrated that, although the United States had the highest publication counts and extensive collaborative networks, its MCP% was relatively low. Similarly, China and Japan also exhibited low MCP% values. In contrast, the United Kingdom, Germany, and Canada exhibited stronger international collaborative initiative, with MCP% values of 62.2, 58.9, and 51.7, respectively.

### 3.4 Analysis of Authors

A total of 16,060 authors contributed to the research on α‐syn aggregation and propagation in PD. To evaluate author productivity and impact, contributors were ranked based on the number of publications (NP), and their h‐index, g‐index and total citations (TC) were also displayed. The top 10 most productive authors are listed in **Table** **1**. Masliah E ranked first with 75 publications, an h‐index of 46, and a g‐index of 75. Outerio TF followed with 71 publications (h‐index = 43, g‐index = 64), while Dobson CM ranked third with 63 publications (h‐index = 43, g‐index = 63). Although Lee VMY did not rank in the top three in terms of publication count, she demonstrated the highest total citations (TC = 10,342), surpassing all other authors on the list.

**TABLE 1:** Top 10 authors contribute to research on aggregation and propagation of α‐syn in PD.

Author (*N* = 16,060)	NP	h_index	g_index	TC
Masliah E	75	46	75	9673
Outeiro TF	71	36	64	4146
Dobson CM	63	43	63	7294
Vendruscolo M	46	29	46	4277
Melki R	46	28	46	3862
Lee VMY	44	35	44	10,342
Luk KC	44	27	44	7732
Trojanowski JQ	42	33	42	9607
Lashuel HA	42	31	42	3644
Zweckstetter M	42	28	42	4385

**FIGURE 3:**
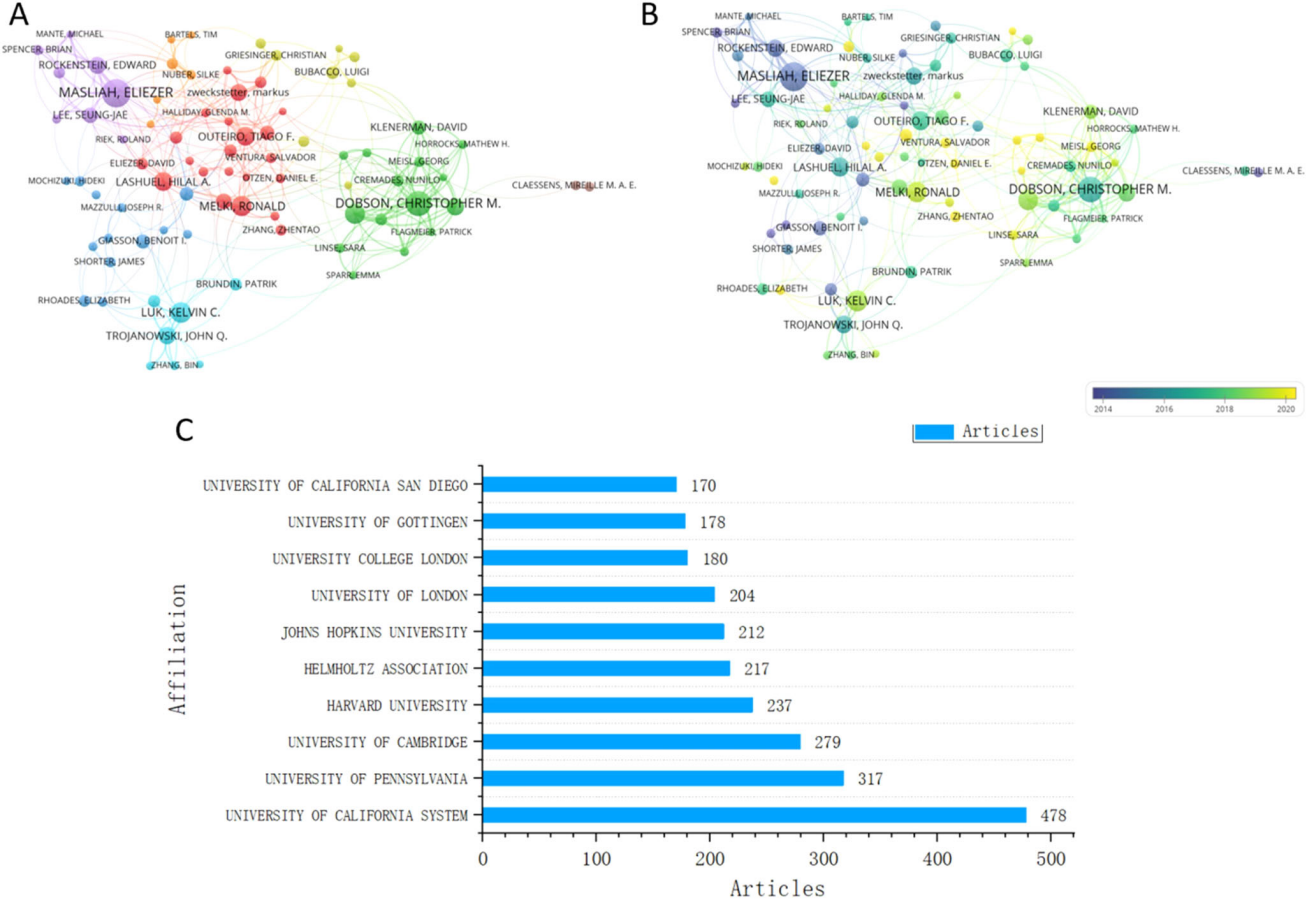
Analysis of authors and affiliations. (A) Co‐authorship network. (B) Co‐authorship overlay map arranged by year. (C) Publications of the top 10 affiliations.

The collaboration networks were visualized to further understand inter‐author relationships (Figure 3A). The network was made up of 81 authors, with a Total Link Strength (TLS) of 1312. Among them, Dobson CM (TLS = 173), Masliah E (TLS = 136), and Vendruscolo M (TLS = 110) were the most central figures, indicating strong collaborative ties with the researchers. In the overlay map, authors with more articles tended to exhibit darker colors like blue or green, while authors with less articles had nodes with lighter colors like yellow, which demonstrated they published articles recently (Figure 3B).

### 3.5 Analysis of Institutions

A total of 1985 institutions were devoted to the specific field and the top 10 institutions with the most articles were exhibited (Figure 3C). These highly contributing institutions were primarily located in Europe and North America. The University of California System emerged as the most prolific institution, with a total of 478 articles, accounting for 15.03% of all included articles, highlighting its significant role in advancing research on α‐syn in PD. The University of Pennsylvania and the University of Cambridge followed, with 317 and 279 publications, ranking second and third, respectively.

### 3.6 Analysis of Journals

All articles were published in 522 academic journals. The top 10 journals with the highest number of publications related to α‐syn aggregation and propagation in PD are listed in **Table** **2**. Science Reports published 131 relative articles, ranking first among all these journals. PLOS One and Proceedings of the National Academy of States of America were second and third with 109 articles and 103 articles relatively. Apart from NP, other metrics were used to assess journals impact, such as total citations (TC), impact factor (IF), and Journal Citation Reports (JCR) rankings. TC of the 10 journals ranged from 840 to 18,438, and IF (2023) fluctuated between 2.9 and 9.4. Proceedings of the National Academy of States of America, which boasted both the highest TC and the highest IF among the 10 journals, was considered high‐quality and influential. According to JCR rankings, the journals listed below were of high scholastic reputations, for they were all classified as Q1 or Q2, reflecting their high academic reputation and relevance in the field.

**TABLE 2:** Top 10 most productive journals.

Source	NP	TC	IF (2023)	JCR
Scientific Reports	131	5161	3.8	Multidisciplinary Sciences (Q1)
PLOS One	109	6405	2.9	Multidisciplinary Sciences (Q1)
Proceedings of the National Academy of Sciences of the United States of America	103	18,438	9.4	Multidisciplinary Sciences (Q1)
International Journal of Molecular Sciences	98	1354	4.9	Biochemistry & Molecular Biology (Q1); Chemistry, Multidisciplinary (Q2)
Journal of Biological Chemistry	95	9486	4	Biochemistry & Molecular Biology (Q2)
Neurobiology of Disease	82	3767	5.1	Neurosciences (Q1)
Acta Neuropathologica Communications	66	2983	6.2	Neurosciences (Q1)
Journal of Neurochemistry	59	2946	4.2	Biochemistry & Molecular Biology (Q2); Neurosciences (Q2)
ACS Chemical Neuroscience	59	1608	4.2	Biochemistry & Molecular Biology (Q2); Chemistry, Medicinal (Q2); Neurosciences (Q2)
NPJ Parkinsons Disease	54	840	6.7	Neurosciences (Q1)

### 3.7 Analysis of Articles

To evaluate the quality of these articles, we compiled the top 10 most cited articles on aggregation and propagation of α‐syn in PD based on TC (**Table** **3**). These highly cited papers were predominantly published in prestigious journals, with publication years fluctuating between 2006 and 2016, indicating that they laid a critical foundation for subsequent research in this area. The article with the highest TC (2380) was “Gut Microbiota Regulate Motor Deficits and Neuroinflammation in a Model of Parkinson's Disease,” authored by Sampson TR and published in Cell in 2016 (Sampson et al. 2016). “Pathological α‐synuclein transmission initiates Parkinson‐like neurodegeneration in nontransgenic mice,” published by Luk KC, was also an influential article of high TC (1864) (Luk et al. 2012). In addition to raw citation counts, normalized total citations were also considered to account for differences in citation practices across disciplines and publication years, allowing for more equitable comparisons. While normalized citation metrics had little effect on the ranking of the top three articles, they did result in noticeable shifts in the positions of the remaining entries. However, due to the date analyzed (2004–2024), the discovery of α‐syn in Lewy bodies was not included (Spillantini et al. 1997).

**TABLE 3:** Top 10 most cited articles.

Article	Journal	Author	PY	TC	TC per year	Normalized TC
Gut Microbiota Regulate Motor Deficits and Neuroinflammation in a Model of Parkinson's Disease	Cell	Sampson TR	2016	2380	238.00	29.16
Pathological α‐Synuclein Transmission Initiates Parkinson‐Like Neurodegeneration in Nontransgenic Mice	Science	Luk KC	2012	1864	133.14	14.11
In Vivo Demonstration That Alpha‐Synuclein Oligomers Are Toxic	Proceedings of the National Academy of Sciences of the United States of America	Winner B	2011	1181	78.73	8.47
Inclusion Formation and Neuronal Cell Death Through Neuron‐to‐Neuron Transmission of Alpha‐Synuclein	Proceedings of the National Academy of Sciences of the United States of America	Desplats P	2009	1165	68.53	6.85
Exogenous α‐Synuclein Fibrils Induce Lewy Body Pathology Leading to Synaptic Dysfunction and Neuron Death	Neuron	Volpicelli‐Daley LA	2011	1140	76.00	8.17
Phosphorylation of Ser‐129 Is the Dominant Pathological Modification of Alpha‐Synuclein in Familial and Sporadic Lewy Body Disease	Journal of Biological Chemistry	Anderson JP	2006	1069	53.45	7.47
Gaucher Disease Glucocerebrosidase and α‐Synuclein Form a Bidirectional Pathogenic Loop in Synucleinopathies	Cell	Mazzulli JR	2011	1032	68.80	7.40
α‐Synuclein Occurs Physiologically as a Helically Folded Tetramer That Resists Aggregation	Nature	Bartels T	2011	1001	66.73	7.18
Ataxin‐2 Intermediate‐Length Polyglutamine Expansions Are Associated With Increased Risk for ALS	Nature	Elden AC	2010	1000	62.50	7.65
α‐Synuclein in Parkinson's Disease	CSH Perspectives in Medicine	Stefanis L	2012	974	69.57	7.37

### 3.8 Analysis of Keywords

**FIGURE 4:**
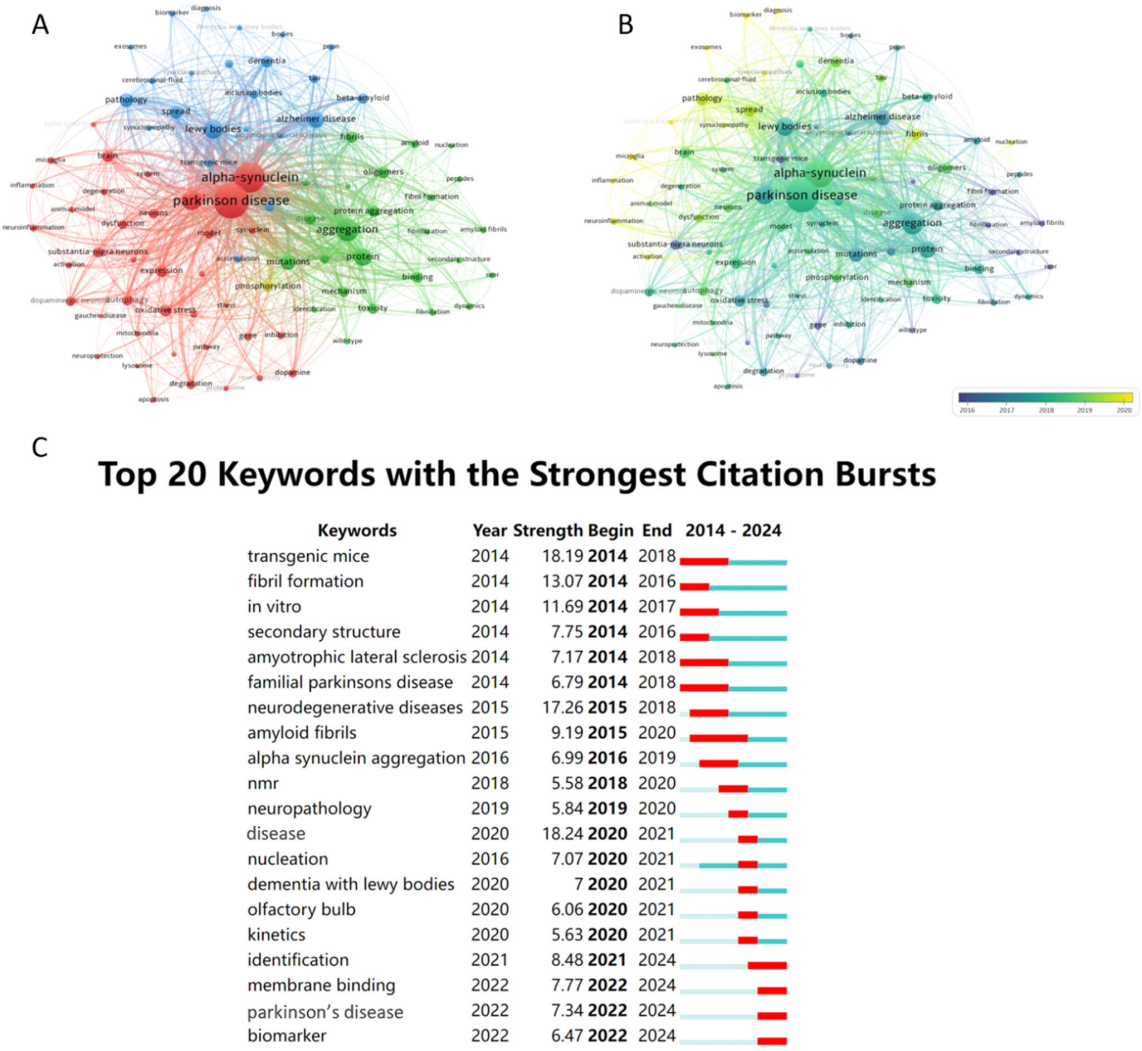
Analysis of keywords. (A) Co‐occurrence keywords network. (B) Co‐occurrence overlay map arranged by year. (C) The top 20 key words with the strongest citation bursts from 2014 to 2024 sorted by the beginning years.

A total of 8006 keywords were extracted from the 3220 articles. After standardization, where synonymous terms and variants were merged into consistent formal expressions, 87 keywords with a frequency of over 50 times occurrences were selected for visual analysis (Figure 4A). The most frequent keywords were “Parkinson's Disease” (2179), “alpha‐synuclein” (1716), and “aggregation” (930), highlighting the central themes of the field.

Cluster 1 (red) focused on the common pathological changes in organs, tissues, and cells in Parkinson's disease. It included keywords such as “alpha‐synuclein,” “dopaminergic neurons,” “degeneration,” “oxidative stress,” “autophagy,” “inflammation,” demonstrating that α‐syn in PD involves complicated pathology. Cluster 2 (green) centered on the molecular‐level alterations during the aggregation of α‐syn. For instance, “peptides” were the basic structure of α‐syn. Under abnormal conditions, “mutations” promoted α‐syn to “aggregation,” and then “oligomers” formed, which led to “toxicity.” Finally, “fibril formation” progressed continuously and PD developed. Cluster 3 (blue) primarily discussed the propagation of α‐syn, highlighting the function of “exosomes” and “inclusion bodies” during the spreading process. “Biomarker” was also involved, which may contribute to diagnosis of PD. Besides, “prion” and “beta‐amyloid,” significant protein in other degenerative diseases, occurred frequently, which allowed us to learn propagation of α‐syn by analogy. Interestingly, only one keywords “phosphorylation,” related to the post‐translational modification mechanism, was classified as Cluster 4 (yellow).

In the overlay map, the color gradient represented the average year of the keywords appearance, providing insight into the temporal evolution of research trends (Figure 4B). The term “aggregation” appeared in blue‐green, whereas “spread” was displayed in yellow‐green, suggesting a recent shift in research focus from α‐syn aggregation to its propagation. Additionally, keywords such as “microglia,” “inflammation,” “exosomes,” and “biomarker” were highlighted in yellow, indicating emerging areas of interest that provided novel and deeper insights into α‐syn pathology.

Furthermore, citation burst analysis, which detects keywords experiencing a sudden surge in citation, was used to identify research hotspots. The top 20 keywords with the strongest citation bursts were primarily concentrated between 2014 and 2024 (Figure 4C). “Fibril formation” (2014–2018), “secondary structure” (2014–2016), and “amyloid fibrils” (2015–2020) suggested that the molecular structure of α‐syn had captured researchers' attention, laying the foundation for further studies on α‐syn. “Neuropathology” (2019–2020) and “dementia with Lewy bodies” (2020–2021) indicated that researchers were committed to investigating the neuropathology of PD. “Olfactory bulb” (2020–2021) demonstrated that research on α‐syn had focused on specific brain regions, which may be linked to the propagation of α‐syn. “Membrane binding” (2022–2024) shifted the focus toward more refined structures, reflecting an evolution in research perspectives that may also be associated with advancements in research techniques. “Biomarker” (2022–2024) was related to PD diagnosis, suggesting that researchers had proposed novel approaches for diagnosing PD.

## 4 Discussion

Overall, the evolving number of publications reveals a clear upward trend in research interest surrounding α‐syn, with rising citation counts further underscoring the scientific impact and recognition of this research area. This is largely attributable to technological advances, like improved imaging and biochemical techniques. For instance, confocal fluorescent microscopes have been widely applied to investigate α‐syn pathology in greater depth (Murray 1992). The increasing global burden of PD is another driving force (Ben‐Shlomo et al. 2024). As the prevalence of PD rises, so too does the urgency to elucidate its underlying mechanisms.

Several countries, especially developed ones, stood out as significant contributors to this research field. Undoubtedly, research funding is the main factor, wealthier nations typically allocate more resources to scientific research, fostering superior research environments and cultivating a larger pool of scientific talent. These factors collectively result in higher‐quality and higher‐quantity outputs. Additionally, many of the leading countries in publication volume face an aging population, which is particularly relevant given PD's prevalence among the elderly (Baker and Petersen 2018). Consequently, the rising incidence of PD in these countries has further fueled national‐level investment and interest in related research. From the perspective of international collaboration, globalization has fostered a highly interconnected research network. Faced with shared global health challenges, cross‐border collaboration is essential for effective problem‐solving. MCP% data further reveal that smaller countries often engage in more international collaboration, likely due to limited domestic resources, such partnerships are essential for these nations to completing scientific projects, making international cooperation a strategic feature of their research ecosystems.

Within the research field, a significant number of highly influential authors and institutions have emerged, primarily concentrated in Western countries. For instance, Virginia Lee, the most cited author, discovered that synthetic misfolded α‐syn can induce disease pathology and neurodegeneration in healthy mice, laying a foundation for subsequent studies on α‐syn propagation (Luk et al. 2012). The University of California System, which published the highest number of articles, has produced many highly cited papers, thanks to its extensive network of affiliated research centers. Notably, the most cited article “Gut Microbiota Regulate Motor Deficits and Neuroinflammation in a Model of Parkinson's Disease” was published by this institution, highlighting the emerging link between PD and the peripheral nervous system (Sampson et al. 2016).

Journal analysis can assist researchers in selecting appropriate publications for literature review. Numerous journals have published a substantial number of α‐syn‐related studies with high citation counts, indicating their influence. According to Journal Citation Reports (JCR), these journals span multiple disciplines, including neuroscience, biochemistry, and molecular biology, underscoring the interdisciplinary nature of α‐syn research and the need for collaboration among scholars from diverse fields. Interestingly, the top 10 journals by number of publications (NP) accounted for only 26.6% of total articles, while over 70% were published in other journals. This suggests that NP alone should not be the sole criterion for journal selection, as lower‐NP journals may also contain highly valuable research. For example, Neuron (IF = 14.1) published only 12 related articles, yet achieved remarkable TC of 4196, surpassing many top 10 NP‐ranked journals. Cell (IF = 45.6), with just 9 relevant articles, included the most highly cited article in this field. These findings emphasize the importance of considering both quantity and quality when assessing journal impact.

Critical analysis of research articles is essential, as high‐impact studies typically feature innovative experimental approaches, advanced methodologies, or groundbreaking conclusions. Researchers can leverage insights from such high‐quality publications to formulate new hypotheses, refine experimental designs, and advance investigations in the field. Among the top 10 articles, some studies focus on post‐translational modifications of α‐syn, proposing that accumulation of normally produced Ser‐129 phosphorylated α‐syn is responsible for the formation of Lewy bodies, which served as a theoretical framework for understanding α‐syn aggregation (Anderson et al. 2006). Others provide compelling evidence that α‐syn propagates between neurons, inducing neurodegeneration and cell death—a pivotal advance in elucidating PD pathogenesis (Desplats et al. 2009; Luk et al. 2012). Furthermore, innovative hypotheses have linked PD to other disorders, highlighting the body as an interconnected system. For instance, deficiency in glucocerebrosidase in Gaucher Disease impairs lysosomal protein degradation, driving α‐syn accumulation (Mazzulli et al. 2011). Emerging data suggest intestinal microbial alterations may trigger PD, implying a gut‐brain axis in neurodegeneration (Sampson et al. 2016). These findings underscore that cross‐disciplinary perspectives are vital for unraveling complex disease mechanisms. In summary, these high‐quality studies not only provide a theoretical foundation for future research but also offer valuable experimental frameworks worthy of emulation by researchers.

**FIGURE 5:**
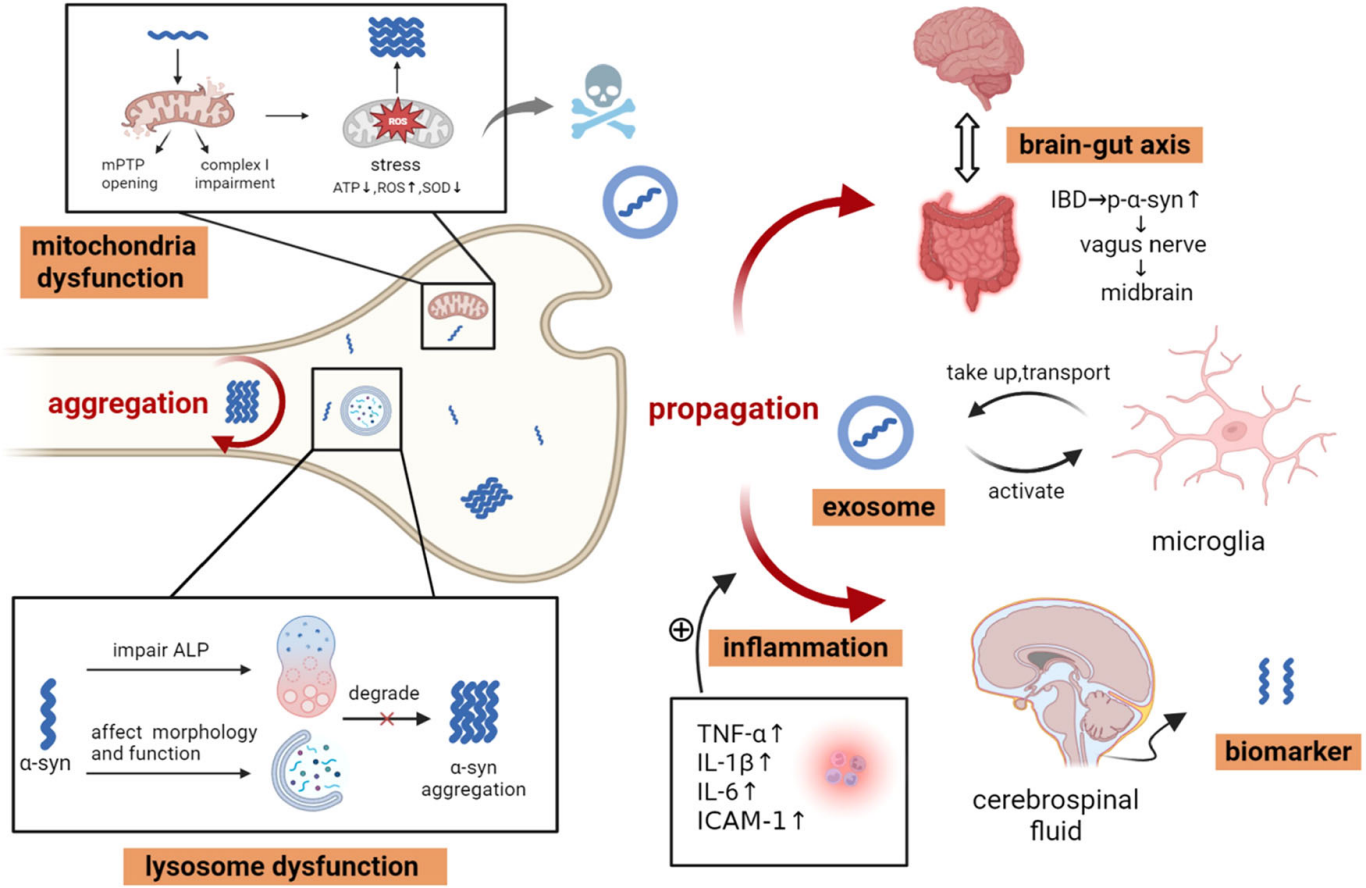
Mechanisms of aggregation and spreading of α‐syn in PD. Abbreviations: ATP, adenosine triphosphate; ROS, reactive oxygen species; SOD, superoxide dismutase; ALP, autophagosome‐lysosome fusion pathway; TNF, tumor necrosis factor; IL, interleukin; ICAM, intercellular cell adhesion molecule; IBD, inflammatory bowel disease.

Keyword clustering analysis revealed two primary research themes: aggregation and propagation. α‐syn aggregation encompasses pathological processes in PD, including α‐syn phosphorylation, oligomer formation, and fibril generation. By integrating findings from Cluster 1, which highlighted various cellular structures, and referencing multiple studies, we identified that subcellular compartments such as mitochondria and lysosomes regulate α‐syn aggregation, a process linked to autophagy. α‐syn propagation, on the other hand, is frequently associated with exosomes and involves the participation of glial cells. Notably, α‐syn propagation is not only implicated in PD progression but also shows strong connections to other diseases, such as inflammatory bowel disease (IBD). From a diagnostic perspective, α‐syn propagation offers potential biomarkers, as clinical detection in the brain is challenging, whereas cerebrospinal fluid (CSF) analysis provides a feasible alternative. Interestingly, the two clusters exhibited significant differences in average publication year (APY), reflecting a recent surge in research focus on α‐syn propagation. This shift underscores an evolving understanding of PD‐no longer viewed solely as a genetically driven protein‐misfolding disorder, but rather as a condition influenced by multi‐system factors. To further clarify α‐syn's pathological mechanisms, we manually consolidated these keywords to mitigate potential inaccuracies from algorithmic clustering, and synthesized them into a mechanistic diagram, enhancing the logical coherence of our discussion (Figure 5).

Mitochondria have drawn considerable attention due to their roles in oxidative phosphorylation and energy metabolism. Mitochondria, as the primary sites of oxidative phosphorylation and cellular energy production, are closely linked to the maintenance of cellular functions. The formation of Lewy bodies is not merely a simple fibrillization process but rather acts as a major driver of neurodegeneration by disrupting cellular functions, inducing mitochondrial damage and dysfunction, and triggering synaptic impairment (Mahul‐Mellier et al. 2020). Cardiolipin, a mitochondrial membrane lipid, plays a critical role in α‐syn protein folding (Choi et al. 2022). In neurons with SNCA mutations, cardiolipin on the mitochondrial surface binds to α‐syn, triggering its oligomerization and subsequent accumulation on the mitochondrial membrane (Ryan et al. 2018). Once α‐syn accumulates excessively on mitochondria, it can translocate into the mitochondrial matrix, which is an irreversible process (Martínez et al. 2018). As α‐syn accumulates, mitochondrial function becomes impaired. The α‐syn aggregates interacting with adenosine triphosphate synthase (ATPase) induce mitochondria permeability transition pore (mPTP) opening, membrane depolarization and loss of phosphorylation capacity, causing mitochondrial swelling and leading to neuronal death (Banerjee et al. 2010; Ludtmann et al. 2018). Furthermore, after α‐syn damages complex I, mitochondrial respiration is inhibited, resulting in oxidative stress manifested by reduced adenosine triphosphate (ATP) production, increased reactive oxygen species (ROS), and decreased superoxide dismutase (SOD) (Ganjam et al. 2019; Scudamore and Ciossek 2018). The consequences of oxidative stress not only include mitochondrial dysfunction but can also exacerbate α‐syn aggregation, suggesting that the relationship between α‐syn aggregation and mitochondrial dysfunction may represent a positive feedback loop where either stimulus can trigger a self‐sustaining process leading to similar outcomes.

Lysosomes are also central to PD pathogenesis through their role in autophagic degradation. α‐syn impairs the autophagosome‐lysosome fusion pathway (ALP), hindering clearance and promoting further aggregation (Korolchuk et al. 2009; Tang et al. 2021). Key regulators include SNARE proteins, TFEB, and AKT/mTORC1 signaling (Decressac et al. 2013; Xiao et al. 2018). α‐syn fibrils can alter lysosomal morphology, function, and distribution (Dilsizoglu Senol et al. 2021). Impaired lysosomal function disrupts α‐syn clearance, causing its accumulation, while the redistribution of lysosomes to the cell periphery facilitates the transfer of damaged lysosomes carrying α‐syn fibrils from donor to recipient cells, promoting α‐syn propagation (Jinn et al. 2017). Additionally, deficiencies in lysosomal enzymes can influence α‐syn aggregation. For example, the lack of glucocerebrosidase (GCase) or α‐galactosidase A (α‐Gal A) in lysosomal storage disorder is often a risk factor for PD. This interplay highlights the critical role of lysosomal dysfunction in PD pathogenesis through impaired α‐syn clearance and intercellular spread (Magalhaes et al. 2016; Mazzulli et al. 2011; Nelson et al. 2018).

According to the overlay map, exosomes have recently emerged as a key mechanism in the propagation of α‐syn. These small extracellular vesicles facilitate the spread of misfolded proteins, a process often linked to microglial involvement. Microglia can be activated by exosomes, primarily through toll‐like receptors (TLRs), particularly TLR2 and TLR4 (Daniele et al. 2015). Once activated, microglia internalize exosomes and efficiently transport them to other neurons, promoting α‐syn spreading (Guo et al. 2020; Xia et al. 2019). Additionally, inflammation plays a critical role in α‐syn propagation. It is reported that tumor necrosis factor (TNF)‐α stimulates lysosomal exocytosis, enhancing α‐syn secretion (Bae et al. 2022). In the periphery, inflammatory bowel disease (IBD) is associated with elevated levels of TNF, interleukin (IL)‐1β, IL‐6, and intercellular adhesion molecule (ICAM)‐1, influenced by gut microbiota (Sampson et al. 2016). This inflammatory milieu promotes α‐syn phosphorylation and aggregation, enabling its spread to the brain via the vagus nerve, which is deemed as brain‐gut axis (Espinosa‐Oliva et al. 2024). Environmental toxins such as lipopolysaccharide (LPS), when inhaled, can trigger olfactory bulb (OB) inflammation, where IL‐1β/IL‐1R1 signaling plays a crucial role in inducing and propagating pathological α‐syn (Niu et al. 2020). Notably, while α‐syn propagation may exacerbate PD, it also offers potential for early diagnosis and intervention. α‐syn can be detected in body fluids, particularly cerebrospinal fluid (CSF), where its presence primarily reflects CNS‐derived α‐syn rather than peripheral sources (Stuendl et al. 2016). This discovery opens new avenues for biomarker development and targeted therapies in PD.

Among the 20 keywords with the most pronounced citation bursts, recent demonstrates a trend toward interdisciplinary integration and enhanced translational potential. Regarding α‐syn aggregation and propagation, the focus has evolved from the intrinsic protein structure to lipid interactions. Notably, the role of membrane binding in α‐syn pathology, whether promotive or inhibitory of PD progression, remains contentious. While some evidence indicates that lipid membranes can nucleate α‐syn amyloid fibril formation, thereby accelerating PD pathogenesis, recent studies further explore PD‐related mutants (e.g., E46K, E35K) through the membrane binding, revealing their propensity for larger multimer formation on bilayers and enhanced liposome‐induced aggregation (Ma et al. 2024). Conversely, other studies propose that attenuated membrane binding may exacerbate neurotoxicity, as exemplified by the V15A mutation which promotes α‐syn aggregation through reduced membrane affinity (Buratti, Fernández, and Zweckstetter 2023; Burré et al. 2015).

The keywords “identification” and “biomarker” reflect a growing emphasis on clinical applications of α‐syn pathology. Current efforts focus on correlating cerebrospinal fluid (CSF) α‐syn levels with PD severity for early diagnosis. Given the presence of α‐syn in healthy individuals, total α‐syn quantification lacks diagnostic specificity. Consequently, researchers produced conformation‐specific α‐syn species—such as α‐syn tetramers or astrocytic extracellular vesicles harboring α‐syn (de Boni et al. 2024; Wang et al. 2023)—to improve diagnostic accuracy. Additionally, differentiating PD from other synucleinopathies (e.g., Multiple System Atrophy) is clinically critical. Pioneering work by Okuzumi et al. identified propagative α‐syn seeds as a serum biomarker (Okuzumi et al. 2023), while Dutta et al. demonstrated that α‐syn levels in plasma‐isolated oligodendrocyte exosomes effectively distinguish PD from MSA (Dutta et al. 2021). These advances hold significant promise for refining PD diagnostics.

The aggregation and propagation of PD involve complex interactions across multiple subcellular compartments and cell types. However, no clinically available treatments currently exist that effectively inhibit α‐syn aggregation, degrade existing aggregates, or prevent intercellular transmission. Although monoclonal antibodies targeting α‐syn, such as Prasinezumab and Cinpanemab, have advanced to clinical trials, their outcomes have been disappointing (Lang et al. 2022; Pagano et al. 2022). This therapeutic impasse necessitates exploration of alternative strategies. Targeting organelles involved in α‐syn pathology may offer new avenues. Particularly promising is targeted protein degradation (TPD), an innovative approach leveraging cellular autophagic machinery to eliminate pathogenic proteins (Luh et al. 2020). Recent advances include Qu et al. designed a targeted peptide, capable of penetrating cell membranes, binding to intracellular α‐syn proteins, and directing the protein complexes to the proteasome for degradation (Qu et al. 2020). Kargbo et al. designed proteolysis‐targeting chimera (PROTAC) compounds with E3 ubiquitin ligase‐binding activity to target α‐syn (Kargbo 2020). Lee et al. developed Autophagy‐Targeting Chimera (AUTOTAC) and discovered that the PD‐Autotac compound ATC161 induces targeted autophagy‐lysosomal degradation of α‐syn aggregates (Lee et al. 2023). Unlike monoclonal antibodies that merely bind targets, TPD mobilizes intrinsic clearance mechanisms (e.g., autophagy‐lysosomal pathways), concurrently mitigating DNA damage and mitochondrial dysfunction. This strategy aligns with emerging research hotspots identified in our study and constitutes a transformative therapeutic paradigm.

In summary, our study holds dual significance at both theoretical and practical levels. Theoretically, we conducted a systematic review of key molecular mechanisms involved in α‐syn aggregation and propagation, providing an in‐depth explanation of the pathological processes underlying PD development. On the practical side, we performed a detailed analysis of publication outputs, collaborative networks among countries, authors, and institutions, as well as the quality of relevant journals and articles, offering valuable reference information for researchers in literature review or manuscript submission. Furthermore, our analysis of research hotspots in this field may guide investigators in selecting appropriate research directions. However, we must acknowledge certain limitations in this study. Our data selection was confined to English articles indexed in WoSCC, potentially excluding relevant studies published elsewhere. Additionally, despite rigorous methodology, data omissions may have occurred during analytical processes due to either human or technical factors. Given the substantial dataset, it was challenging to conduct exhaustive analyses of all information, and subjective selection criteria might have led to incomplete coverage. For instance, in keyword analysis, we prioritized terms deemed most significant, possibly overlooking certain molecular mechanisms worthy of exploration. Despite these limitations, our study provides researchers with meaningful insights and holds potential clinical applications.

## 5 Conclusion

This study provides both theoretical and practical contributions. Theoretically, it offers a comprehensive review of the mechanisms underlying α‐syn aggregation and propagation over the period from 2005 to 2024, based on an analysis of 3220 articles retrieved from WoSCC. The findings demonstrate a consistent rise in publication volume, reflecting growing research interest and scientific attention in this field. The United States ranks first in research output, followed by China and United Kingdom, with international collaboration serving as a key driver of progress. Prominent journals such as Scientific Reports have significantly contributed to the high‐impact studies. Thematic clustering of keywords revealed two major research focuses: α‐syn aggregation, closely associated with mitochondrial dysfunction and lysosomal impairment, and α‐syn propagation pathways, particularly through exosome‐mediated pathways. Emerging research trends emphasize the roles of microglia activation, gut‐brain axis interactions, and the development of biomarkers for early diagnosis of PD. Overall, this study provides a comprehensive and up‐to‐date understanding of α‐syn research, offering meaningful guidance for future investigations and development of potential therapeutic strategies.

### 5.1 Limitations

While this study focused on α‐syn aggregation and propagation in PD, two key limitations warrant emphasis. First, the analysis scope was inherently constrained by exclusive reliance on the Web of Science Core Collection (WoSCC) database. Although WoSCC indexes most high‐quality peer‐reviewed publications, it remains possible that seminal works beyond its coverage were omitted. Second, the application of the “All Open Access” filter may exclude significant non‐Open Access studies. To mitigate these constraints, future research will adopt a multi‐database approach to validate and extend the current findings.

## Author Contributions

X.Z. had unrestricted access to the complete dataset in the study and bears accountability for maintaining the integrity and precision of both data and analyses. Concept and design: Q.W. Acquisition, analysis, and interpretation of data: X.Z., Y.M., G.G. Drafting of the manuscript: X.Z. Revision of the manuscript: X.Z., G.G., and Q.W. Statistical analysis: X.Z. and Y.M. Administrative, technical, or material support: J.H., G.G., and Q.W. Supervision: Q.W.

## Funding

This study is supported by National Natural Science Foundation of China (82101486 and 82371426 to Q.W., 82471448 to G.G.), Science and Technology Commission of Shanghai Municipality (STCSM) grant (23ZR1467900 to Q.W.), Ningxia Hui Autonomous Region Key Research and Development Project(2022BFH02012 to Q.W.), Shanghai Fourth People's Hospital Affiliated to Tongji University School of Medicine (sykyqd02301 and sykyqd02302 to Q.W.), the Fundamental Research Funds for the Central Universities (Q.W., G.G.), Tongji University Medicine‐X Interdisciplinary Research Initiative (No. 2025‐0650‐ZD‐06 to Q.W.), Shanghai Pujiang Program (21PJ1412100 to Q.W.), and Shanghai Rising‐Star Program (23YF1450300 to Y.M.).

## Conflicts of Interest

The authors declare no competing interests.

## Peer Review

The peer review history for this article is available at https://publons.com/publon/10.1002/brb3.71023

## Data Availability

The data that support the findings of this study are available from the corresponding author upon request.
