# Correction to “Aggregation and Propagation of α‐Synuclein in Parkinson's Disease: A Bibliometric Perspective”

**DOI:** 10.1002/brb3.71156

**Published:** 2026-02-08

Zhang X, Ma Y, Gao G, Wu Q. Aggregation and Propagation of α‐Synuclein in Parkinson's Disease: A Bibliometric Perspective. Brain Behav. 2025;15(11):e71023. doi:10.1002/brb3.71023

In sections “3.2 Distribution of Countries” and “3.3 Collaboration Among Countries,” as well as in Figure 2, all instances of “countries” should be corrected to “countries/regions.”

In paragraph 1 of the “3.3 Collaboration Among Countries” section, the text “A national collaboration network illustrated these cooperative relationships (Figure 2E).” was incorrect. This should have read: “An international collaboration network illustrated these cooperative relationships (Figure 2E).”

We apologize for this error.

